# Vitamin D Levels During Pregnancy and Dental Caries in Offspring

**DOI:** 10.1001/jamanetworkopen.2025.46166

**Published:** 2025-12-02

**Authors:** Nuo Xu, Zexin Chen, Boya Wang, Yiwen Qiu, Xialidan Alifu, Haibo Zhou, Haoyue Cheng, Ye Huang, Libi Zhang, Hui Liu, Lina Yu, Danqing Chen, Yunxian Yu

**Affiliations:** 1 Department of Public Health, and Department of Anesthesiology, Second Affiliated Hospital of Zhejiang University School of Medicine, Hangzhou, China; 2 Department of Epidemiology and Health Statistics, School of Public Health, School of Medicine, Zhejiang University, Hangzhou, China; 3 Clinical Research Center, Sir Run Run Shaw Hospital, School of Medicine, Zhejiang University, Hangzhou, China; 4 Zhejiang Key Laboratory of Pain Perception and Neuromodulation, Hangzhou, China; 5 Department of Obstetrics, Women’s Hospital, School of Medicine, Zhejiang University, Hangzhou, China

## Abstract

**Importance:**

Maternal vitamin D level during pregnancy has been repeatedly reported to be associated with early childhood caries (ECCs) in offspring, yet the conclusions remain inconsistent.

**Objective:**

To evaluate the association between maternal vitamin D status in different trimesters during pregnancy and offspring dental caries.

**Design, Setting, and Participants:**

This prospective cohort study was based on the Zhoushan Pregnant Women Cohort, which enrolled pregnant women between August 2011 to May 2021, and followed up on their offspring until November 2022, at Zhoushan Maternal and Child Health Hospital in Zhoushan, Zhejiang Province, China. Data were analyzed from October 2024 to April 2025.

**Exposures:**

Maternal plasma 25-hydroxyvitamin D (25[OH]D) levels measured in the first, second, and third trimesters and vitamin D deficiency (VDD).

**Main Outcomes and Measures:**

The primary outcome was offspring ECCs. Secondary outcomes included the decayed, missing, or filled teeth (dmft, with lowercase term denoting primary, instead of permanent, dentition) index and caries rate (the ratio of dmft to number of erupted teeth).

**Results:**

The cohort included 4109 mother-offspring pairs (maternal median [IQR] age, 29.0 [27.0-32.0] years; offspring gestational age at birth, 39.0 [38.0-40.0] weeks; 2121 males [51.6%]), of whom 960 children had ECCs and 3149 did not. Higher maternal 25(OH)D levels were associated with reduced odds of ECCs in offspring (first trimester odds ratio [OR], 0.98 [95% CI, 0.97-0.99], false discovery rate [FDR]-adjusted *P* = .009; second trimester OR, 0.98 [95% CI, 0.97-0.99], FDR-adjusted *P* = .001; third trimester OR, 0.99 [95% CI, 0.98-1.00], FDR-adjusted *P* = .009), while Cox proportional hazards regression models showed protective benefits of 25(OH)D levels against ECC risk throughout all trimesters. Categorical analyses suggested elevated odds of ECCs in offspring of mothers with vitamin D insufficiency, VDD, or severe VDD compared with the vitamin D sufficiency group, although the statistical significance of some associations was attenuated after FDR correction. Moreover, higher maternal 25(OH)D levels (μg/mL) were associated with lower dmft scores (third trimester: β [SE] = −9.97 [3.97]; *P* = .01) and caries rate (third trimester: β [SE] = −50.87 [19.78]; *P* = .01). Generalized estimation equation models also confirmed the inverse associations.

**Conclusions and Relevance:**

In this cohort study, maternal 25(OH)D levels throughout pregnancy were inversely associated with odds of offspring ECCs. These findings support the potential benefit of vitamin D supplementation before or during pregnancy in reducing the risk and severity of childhood dental caries.

## Key Points

**Question:**

Are maternal vitamin D levels associated with early dental caries (ECCs) in offspring, and is there variation by pregnancy trimester?

**Findings:**

In this cohort study of 4109 mother-offspring pairs, lower levels of maternal 25-hydroxyvitamin D during pregnancy, particularly in the mid- to late-trimesters, were associated with increased odds of ECCs.

**Meaning:**

These findings suggest that integrating vitamin D screening and supplementation into routine prenatal care during and even before pregnancy may help reduce the odds of childhood ECC.

## Teaser

This cohort study examines the association between vitamin D sufficiency or deficiency in pregnant mothers and the development and severity of dental caries in their offspring.

## Introduction

Early childhood caries (ECC), defined as the presence of 1 or more cavities or noncavitated lesions, missing (due to caries), or filled tooth surfaces in children up to 71 months of age,^1^ is an important public health concern. An evaluation across 193 United Nations countries from 2007 to 2017 indicated a mean ECC prevalence of 23.8% in children younger than 3 years and 57.3% in children aged 3 to 6 years.^2^ The 2015 Fourth National Oral Health Survey in China revealed a progressive increase in dental caries prevalence among Chinese preschoolers, reaching 71.9% in those aged 5 years.^3^ Moreover, ECCs remain the leading oral disease affecting children’s oral health as well as the most common chronic noncommunicable disease in children.^4^ ECCs can impact children’s physiological functions, such as chewing, and secondary pulp and apical diseases, appearance, and even academic performance,^5,6,7^ severely affecting their oral and general health and increasing the economic burden on family and society.^8^ Hence, preventing and controlling ECCs is a critical public health challenge that requires worldwide attention and solutions.

The developmental origins of ECCs can be traced back to the prenatal and early postnatal periods, mainly through mechanisms involved in tooth mineralization.^9^ Some studies have suggested that the 13th to 17th gestational weeks are the critical period for the mineralization of maxillary anterior teeth, and most primary teeth begin to mineralize from the second to the third trimester.^10,11,12^ During these periods, vitamin D is essential for maintaining calcium and phosphate homeostasis, regulating phosphate-calcium homeostasis during enamel formation and remineralization after enamel eruption.^13,14^ Maternal vitamin D levels can affect primary tooth mineralization; for example, functional vitamin D receptors (VDRs) are present in the ameloblasts and odontoblasts at sufficient levels to support enamel matrix formation and prismatic organization.^15,16^ Despite global recommendations to maintain maternal 25-hydroxyvitamin D (25[OH]D) levels above 30 ng/mL (to convert to nanomole per liter, multiply by 2.496) during pregnancy, vitamin D deficiency is still prevalent, which can lead to developmental enamel defects and increased susceptibility to caries in offspring.^17,18^ Therefore, understanding the role of maternal vitamin D status during pregnancy in children’s dental health is of great importance.

In recent decades, the association between maternal vitamin D during pregnancy and the risk of ECCs in offspring has been widely reported.^19,20,21^ Existing evidence of randomized clinical trials appeared insufficient to support that maternal vitamin D supplementation during pregnancy confers protection against ECCs in offspring,^22,23^ primarily due to limitations in sample sizes and study designs (eg, vitamin D supplementation initiated only in the second and third trimesters). Furthermore, observational studies have yielded inconsistent results regarding the association between maternal vitamin D status and offspring ECCs,^24,25^ which may be attributed to substantial heterogeneity across studies and the nonuniform gestational week of maternal blood sampling and age at child dental examination. Thus, in this cohort study, we aimed to evaluate the association between maternal vitamin D status in different trimesters during pregnancy and offspring dental caries.

## Methods

### Study Design and Participants

This prospective cohort study was based on the Zhoushan Pregnant Women Cohort (ZPWC). In the ZPWC, pregnant women were enrolled at Zhoushan Maternal and Child Health Hospital in Zhoushan, Zhejiang Province, China, between August 2011 to May 2021, and their offspring were followed up until November 2022. The Zhejiang University School of Medicine Institutional Review Board approved this cohort study. All participants provided written informed consent. The study was conducted according to the guidelines of the Declaration of Helsinki.^26^ We followed the Strengthening the Reporting of Observational Studies in Epidemiology (STROBE) reporting guideline.

The inclusion and exclusion criteria of the ZPWC have been described previously.^27^ In brief, pregnant women were included if they met the following criteria: (1) age between 18 and 45 years, (2) singleton pregnancy, (3) gestational weeks of first antenatal examination between 5 and 13 weeks, (4) at least 1 plasma vitamin D measurement during pregnancy, and (5) offspring with 1 or more oral health examination records between 12 and 71 months of age. The exclusion criteria were (1) maternal body mass index (BMI; calculated as weight in kilograms divided by height in meters squared) higher than 40 or lower than 15 at the first prenatal visit, (2) gestational weeks of delivery longer than 45 weeks or earlier than 28 weeks, (3) data on children that could not be linked to the maternal dataset. The participant flowchart and follow-up status are presented in eFigure 1 and eTable 1 in Supplement 1.

### Information and Blood Sample Collection

A face-to-face interview by trained nurses was conducted with pregnant women who enrolled in the ZPWC to collect information on sociodemographic characteristics, lifestyle, and health status in the first trimester (8th to 14th gestational weeks). Follow-up visits were then conducted at the second trimester (24th to 28th gestational weeks), third trimester (32nd to 36th gestational weeks), and 42nd-day post partum, with corresponding questionnaires administered respectively. Additionally, fasting venous blood sample (5 mL) from pregnant women was collected at each visit, centrifuged at 4 °C, and separated into plasma and white blood cells for storage at −80 °C until use. Children born to mothers enrolled in the ZPWC underwent their first follow-up visit at 42 days of age. Subsequent follow-up visits were conducted every 6 months from 6 to 30 months of age and annually from 3 to 5 years of age (ie, 36-47, 48-59, 60-71 months). At each visit, noninvasive examinations were performed, such as physical growth assessments (eg, spine, vision, and oral health), neurodevelopmental evaluations, dietary surveys, and illness records.

### Measurement of Plasma 25(OH)D

The plasma concentrations of 25-hydroxyvitamin D_2_ (25[OH]D_2_) and 25-hydroxyvitamin D_3_ (25[OH]D_3_) were measured by liquid chromatography-tandem mass spectrometry (Waters Corporation). The total 25(OH)D concentration was calculated as the sum of 25(OH)D_2_ and 25(OH)D_3_, reported in ng/mL with a detection lowest limit of 1 ng/mL.

### Exposure and Outcomes

According to the Endocrine Society Clinical Practice Guideline, plasma 25(OH)D less than 20 ng/mL was defined as vitamin D deficiency (VDD).^28^ Due to the lack of unified standards for VDD, we classified 25(OH)D level into 4 groups for sensitivity analysis: severe VDD (<12 ng/mL), VDD (≥12 to <20 ng/mL), vitamin D insufficiency (≥20 to <30 ng/mL), and vitamin D sufficiency (≥30 ng/mL).

Oral health examinations for children younger than 6 years were conducted by trained pediatric dentists following standardized clinical protocol.^29^ The examinations included visual inspection of all erupted primary teeth under adequate lighting, with teeth cleaned using wet gauze or cotton rolls when necessary. The decayed, missing, or filled teeth (dmft, with lowercase term denoting primary, instead of permanent, dentition) index was used to record caries experience, ranging from 0 to 20. Each tooth was assessed for the presence of cavitated or noncavitated carious lesions, restorations, and extractions due to caries. Caries rate was calculated as the ratio of dmft to the number of erupted teeth. ECCs were defined as a dmft score of 1 or higher.^30^ To account for the potential implication of increasing child age for tooth eruption and caries progression, both original and age-standardized dmft score and caries rate were used as continuous outcomes. The offspring ECCs were the primary outcome in this study. Secondary outcomes were dmft score and caries rate.

### Statistical Analysis

Descriptive statistics are presented as median (IQR) for continuous variables (due to all continuous presented non-normal distribution) and as frequency (%) for categorical variables, and comparisons between groups were performed using the Wilcoxon rank-sum and χ^2^ tests, respectively. For the single-time point data (last dental examination record of offspring younger than 6 years), restricted cubic spline models were used to explore the dose-response association and the potential nonlinear relationship between maternal 25(OH)D levels in different trimesters and all 3 offspring caries outcomes.

For the primary outcome of ECC, we used generalized linear regression models to explore the association between maternal vitamin D status in different trimesters and offspring ECCs. Model 1 was adjusted for maternal age, smoking status, parity, educational level, BMI, and gestational age and season of 25(OH)D test as well as offspring gestational age at birth, gender, birth weight, and feeding pattern. Model 2 was additionally adjusted for offspring age at dental caries detection based on model 1. Cox proportional hazards regression models were used to analyze the time to incident ECC from age 1 to 6 years. Event time was defined as the age at first ECC diagnosis, with censoring at the age of the last dental examination for offspring without ECC. Benjamini-Hochberg false discovery rate (FDR) method was applied to adjust for multiple comparisons for all analyses of ECCs.

For secondary outcomes, multiple linear regression was used to explore the association between maternal vitamin D status in different trimesters with offspring dmft score and caries rate. Model 1 used original dmft score and original caries rate as outcomes. Model 2 used age-standardized dmft score and age-standardized caries rate as outcomes. Both models were adjusted for the same covariates as the ECC models. In addition, for the repeated measurement data (all of the oral examination records of offspring younger than 6 years), generalized estimation equation (GEE) models were used. To improve the interpretability of the regression coefficients, 25(OH)D levels were rescaled from ng/mL to μg/mL (dividing by 1000) for all analyses of dmft score and caries rate.

All analyses were performed from October 2024 to April 2025 in R, version 4.3.2 (R Project for Statistical Computing). Two-sided *P* < .05 was considered to be statistically significant. Additional methodological details are described in the eMethods in Supplement 1.

## Results

### Participant Characteristics

A total of 4109 mother-offspring pairs (maternal median [IQR] age, 29.0 [27.0-32.0] years; offspring gestational age at birth, 39.0 [38.0-40.0] weeks; 2121 males [51.6%] and 1988 females [48.4%]) participated in this study, and their baseline sociodemographic characteristics stratified by offspring ECCs are shown in Table 1. At the time of their last dental examination, 960 offspring (23.4%) aged 1 to 6 years had ECCs. Compared with offspring without ECC (n = 3149), those with ECCs were more likely to be older and have higher gestational age at birth and birth weight, while their mothers were more likely to be younger, multiparous, and have lower educational levels. The distribution of ECC indicators (dmft score, caries rate, and ECC status) between age groups is provided in eTable 2 in Supplement 1. It showed that the rate of ECC increased with age growth.

**Table 1.** Basic Characteristics of Mother and Offspring (N = 4109)

Variables	Participants, No. (%)		*P* value
	Without ECCs (n = 3149)	With ECCs (n = 960)	
Maternal			
Age, median (IQR), y	29.0 (27.0-32.0)	28.0 (26.0-31.0)	.01
Prepregnancy BMI, median (IQR)	20.8 (19.3-22.8)	21.0 (19.2-23.3)	.34
Educational level			
≤High school	264 (8.4)	120 (12.5)	<.001
High school diploma	485 (15.4)	201 (20.9)	
≥College degree	2400 (76.2)	639 (66.6)	
Smoking status			
No	3135 (99.6)	953 (99.3)	.41
Yes	14 (0.4)	7 (0.7)	
Parity			
Primipara	2242 (71.2)	631 (65.7)	.001
Multipara	907 (28.8)	329 (34.3)	
Offspring			
Gestational age at birth, median (IQR), wk	39.0 (38.0-40.0)	39.0 (38.0-40.0)	.03
Birth weight, median (IQR), kg	3.3 (3.0-3.6)	3.4 (3.1-3.6)	.02
Age at dental examination, median (IQR), mo	42.0 (36.0-57.0)	58.0 (50.0-64.0)	<.001
dmft Score, median (IQR)	0.0 (0.0-0.0)	2.0 (2.0-4.0)	<.001
Caries rate, median (IQR), %^a^	0.0 (0.0-0.0)	10.5 (10.0-21.1)	<.001
Gender			
Male	1640 (52.1)	481 (50.1)	.30
Female	1509 (47.9)	479 (49.9)	
Feeding pattern			
Full-breastfeeding	50 (1.6)	24 (2.5)	.05
Partial-breastfeeding	2941 (93.4)	876 (91.2)	
Full-formula	158 (5.0)	60 (6.2)	

Abbreviations: BMI, body mass index (calculated as weight in kilograms divided by height in meters squared); dmft, decayed, missing, or filled teeth; ECCs, early childhood caries.

^a^ Caries rate is the proportion of caries in the number of teeth. Caries-related characteristics of offspring (age at dental examination, dmft score, and caries rate) were assessed at the final follow-up examination.

Compared with offspring without ECCs, those with ECCs had median (IQR) maternal 25(OH)D levels that were significantly lower during the second trimester (28.7 [21.2-36.1] vs 26.9 [18.8-34.3] ng/mL; *P* = .001) and the third trimester (30.6 [21.4-39.6] vs 26.1 [17.3-34.7] ng/mL; *P* = .001), with no difference in the first trimester (Figure; eTable 3 in Supplement 1). Consistently, the VDD rate was significantly higher in the ECC group at the second trimester (21.4% vs 27.5%; *P* = .01) and the third trimester (21.5% vs 31.8%; *P* = .001), with no difference in the first trimester.

**Figure.**
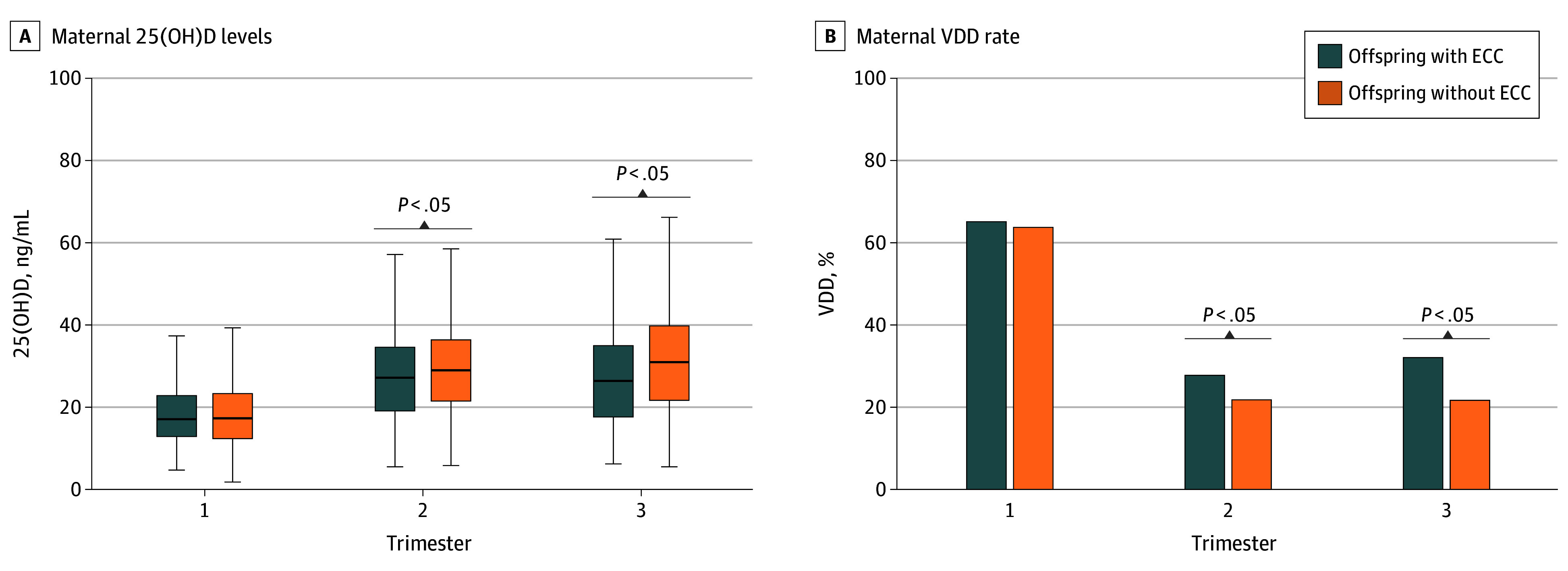
Comparison of Maternal Vitamin D Status During Pregnancy Between Offspring With and Without Early Childhood Caries (ECCs) A, Upper and lower ends of the boxes represent the 75th and 25th percentiles, respectively; horizontal lines inside boxes represent the medians; and whiskers represent the minimum and maximum values within 1.5 IQR. 25(OH)D indicates 25-hydroxyvitamin D, and VDD indicates vitamin D deficiency.

### Association of Maternal Vitamin D Status With Offspring ECCs

No significant nonlinear associations were observed between maternal 25(OH)D levels and offspring dmft score, caries rate, and ECCs across 3 trimesters in the restricted cubic spline models (all *P* for nonlinear >.05) (eFigures 2 and 3 in Supplement 1). The smoothed curves suggested inverse associations in the second and third trimesters, where higher 25(OH)D levels were associated with lower estimated odds of dental outcomes.

As shown in Table 2, maternal 25(OH)D levels (continuous variable) were inversely associated with offspring ECC risk in all trimesters (first trimester odds ratio [OR], 0.98 [95% CI, 0.97-0.99], FDR-adjusted *P* = .009; second trimester OR, 0.98 [95% CI, 0.97-0.99], FDR-adjusted *P* = .001; third trimester OR, 0.99 [95% CI, 0.98-1.00], FDR-adjusted *P* = .009). However, there were no associations between maternal VDD (binary variable) during pregnancy and offspring ECCs. But while 25(OH)D level was classified into 4 categories, compared with vitamin D sufficiency (≥30 ng/mL), lower vitamin D status showed increased odds of ECCs across trimesters. Although significant associations were observed only in the third trimester for vitamin D insufficiency (OR, 1.44; 95% CI, 1.10-1.88) and severe VDD (OR, 1.63; 95% CI, 1.02-2.58), the pattern was similar in the first and second trimesters.

**Table 2.** Association Between Maternal Vitamin D Status in Different Trimesters and Offspring Early Childhood Caries

Vitamin D status by trimester	Offspring, No. (%)		Model 1^a^		Model 2^b^	
	Without ECCs	With ECCs	OR (95% CI)	FDR-adjusted *P* value	OR (95% CI)	FDR-adjusted *P* value
**25(OH)D level, ng/mL**						
First trimester	2521 (76.88)	758 (23.12)	1.00 (0.99-1.01)	.55	0.98 (0.97-0.99)	.009
Second trimester	1702 (81.24)	393 (18.76)	0.99 (0.98-0.997)	.02	0.98 (0.97-0.99)	.001
Third trimester	1543 (75.42)	503 (24.58)	0.98 (0.97-0.99)	.003	0.99 (0.98-1.00)	.009
**2 Categories of 25(OH)D levels, %**						
First trimester						
Vitamin D sufficiency: ≥20 ng/mL	905 (77.3)	1616 (76.7)	1 [Reference]	NA	1 [Reference]	NA
VDD: <20 ng/mL	266 (22.7)	492 (23.3)	1.05 (0.88-1.26)	.59	1.18 (0.97-1.43)	.17
Second trimester						
Vitamin D sufficiency: ≥20 ng/mL	1337 (82.4)	365 (77.2)	1 [Reference]	NA	1 [Reference]	NA
VDD: <20 ng/mL	285 (17.6)	108 (22.8)	1.28 (0.97-1.67)	.17	1.26 (0.94-1.67)	.17
Third trimester						
Vitamin D sufficiency: ≥20 ng/mL	1211 (77.9)	332 (67.5)	1 [Reference]	NA	1 [Reference]	NA
VDD: <20 ng/mL	343 (22.1)	160 (32.5)	1.28 (0.99-1.63)	.17	1.16 (0.89-1.51)	.32
**4 Categories of 25(OH)D levels, %**						
First trimester						
Vitamin D sufficiency: ≥30 ng/mL	214 (8.5)	58 (7.7)	1 [Reference]	NA	1 [Reference]	NA
Vitamin D insufficiency: ≥20 to <30 ng/mL	616 (24.4)	164 (21.6)	1.09 (0.78-1.53)	.75	1.35 (0.95-1.93)	.19
VDD: ≥12 to <20 ng/mL	1000 (39.7)	328 (43.3)	1.19 (0.87-1.66)	.41	1.47 (1.05-2.07)	.10
Severe VDD: <12 ng/mL	691 (27.4)	208 (27.4)	0.98 (0.70-1.39)	.91	1.50 (1.04-2.20)	.10
Second trimester						
Vitamin D sufficiency: ≥30 ng/mL	774 (45.5)	146 (37.2)	1 [Reference]	NA	1 [Reference]	NA
Vitamin D insufficiency: ≥20 to <30 ng/mL	83 (4.9)	27 (6.9)	1.26 (0.97-1.63)	.10	1.37 (1.04-1.81)	.07
VDD: ≥12 to <20 ng/mL	282 (16.6)	81 (20.6)	1.40 (1.02-1.93)	.07	1.43 (1.02-2.01)	.07
Severe VDD: <12 ng/mL	563 (33.1)	139 (35.4)	1.54 (0.92-2.53)	.10	1.58 (0.91-2.67)	.10
Third trimester						
Vitamin D sufficiency: ≥30 ng/mL	800 (51.8)	187 (37.2)	1 [Reference]	NA	1 [Reference]	NA
Vitamin D insufficiency: ≥20 to <30 ng/mL	69 (4.5)	44 (8.7)	1.41 (1.09-1.81)	.02	1.44 (1.10-1.88)	.02
VDD: ≥12 to <20 ng/mL	263 (17.0)	116 (23.1)	1.41 (1.05-1.89)	.03	1.31 (0.96-1.79)	.09
Severe VDD: <12 ng/mL	411 (26.6)	156 (31.0)	1.85 (1.18-2.87)	.02	1.63 (1.02-2.58)	.047

Abbreviations: 25(OH)D, 25-hydroxyvitamin D; ECC, early childhood caries; FDR, false discovery rate; NA, not applicable; OR, odds ratio; VDD, vitamin D deficiency.

SI conversion factor: To convert micrograms per milliliter to nanomoles per liter, multiply by 2.496.

^a^ Model 1 was adjusted for maternal age, smoking status, parity, educational level, body mass index, and gestational age and season of 25(OH)D test as well as offspring gestational age at birth, gender, birth weight, and feeding pattern.

^b^ Model 2 was additionally adjusted for age of offspring caries detection.

In terms of Cox proportional hazards regression models, higher maternal 25(OH)D level during pregnancy was associated with reduced hazard of ECC in offspring aged 1 to 6 years (first trimester hazard ratio [HR], 0.99 [95% CI, 0.98-1.00], FDR-adjusted *P* = .02; second trimester HR, 0.98 [95% CI, 0.98-0.99], FDR-adjusted *P* = .001; third trimester HR, 0.99 [95% CI, 0.98-1.00], FDR-adjusted *P* = .03) (Table 3). However, there were no associations of maternal VDD (binary variable) in each trimester with offspring ECCs. For 4 categorical variables, compared with the vitamin D sufficiency group (≥30 ng/mL), significantly higher hazards of ECC were observed in offspring of mothers with vitamin D insufficiency in the second trimester (HR, 1.37; 95% CI, 1.10-1.71; FDR-adjusted *P* = .02) and those with VDD (HR, 1.30, 95% CI, 1.01-1.67; FDR-adjusted *P* = .049) or severe VDD (HR, 1.37; 95% CI, 1.04-1.79; FDR-adjusted *P* = .047) in the first trimester. Other VDD groups had elevated but nonsignificant HRs.

**Table 3.** Association Between Maternal Vitamin D Status at Different Trimesters and Offspring ECCs in Cox Proportional Hazards Regression Models^a^

Vitamin D status by trimester	Offspring, No. (%)		HR (95% CI)	FDR-adjusted *P* value
	Without ECCs	With ECCs		
**25(OH)D level, ng/mL**				
First trimester	2393 (73.0)	886 (27.0)	0.99 (0.98-1.00)	.02
Second trimester	1650 (78.8)	445 (21.2)	0.98 (0.98-0.99)	.001
Third trimester	1463 (71.5)	583 (28.5)	0.99 (0.98-1.00)	.03
**2 Categories of 25(OH)D levels**				
First trimester				
Vitamin D sufficiency: ≥20 ng/mL	862 (73.6)	309 (26.4)	1 [Reference]	NA
VDD: <20 ng/mL	1531 (72.6)	577 (27.4)	1.11 (0.96-1.28)	.40
Second trimester				
Vitamin D sufficiency: ≥20 ng/mL	1290 (79.5)	332 (20.5)	1 [Reference]	NA
VDD: <20 ng/mL	360 (76.1)	113 (23.9)	1.01 (0.81-1.28)	.90
Third trimester				
Vitamin D sufficiency: ≥20 ng/mL	1159 (74.6)	395 (25.4)	1 [Reference]	NA
VDD: <20 ng/mL	304 (61.8)	188 (38.2)	1.11 (0.92-1.34)	.40
**4 Categories of 25(OH)D levels**				
First trimester				
Vitamin D sufficiency: ≥30 ng/mL	204 (75.0)	68 (25.0)	1 [Reference]	NA
Vitamin D insufficiency: ≥20 to <30 ng/mL	658 (73.2)	241 (26.8)	1.26 (0.97-1.63)	.08
VDD: ≥12 to <20 ng/mL	945 (71.2)	383 (28.8)	1.30 (1.01-1.67)	.049
Severe VDD: <12 ng/mL	586 (75.1)	194 (24.9)	1.37 (1.04-1.79)	.047
Second trimester				
Vitamin D sufficiency: ≥30 ng/mL	751 (81.6)	169 (18.4)	1 [Reference]	NA
Vitamin D insufficiency: ≥20 to <30 ng/mL	539 (76.8)	163 (23.2)	1.37 (1.10-1.71)	.02
VDD: ≥12 to <20 ng/mL	280 (77.1)	83 (22.9)	1.12 (0.85-1.48)	.41
Severe VDD: <12 ng/mL	80 (72.7)	30 (27.3)	1.44 (0.93-2.23)	.15
Third trimester				
Vitamin D sufficiency: ≥30 ng/mL	765 (77.5)	222 (22.5)	1 [Reference]	NA
Vitamin D insufficiency: ≥20 to <30 ng/mL	394 (69.5)	173 (30.5)	1.13 (0.92-1.39)	.30
VDD: ≥12 to <20 ng/mL	244 (64.4)	135 (35.6)	1.13 (0.90-1.43)	.30
Severe VDD: <12 ng/mL	60 (53.1)	53 (46.9)	1.35 (1.00-1.82)	.16

Abbreviations: 25(OH)D, 25-hydroxyvitamin D; ECCs, early childhood caries; FDR, false discovery rate; HR, hazard ratio; NA, not applicable; VDD, vitamin D deficiency.

SI conversion factor: To convert micrograms per milliliter to nanomoles per liter, multiply by 2.496.

^a^ Vitamin D status at each trimester was calculated in different models. The Cox proportional hazards regression models were adjusted for maternal age, smoking status, parity, educational level, body mass index, and gestational age and season of 25(OH)D test as well as offspring gestational age at birth, gender, birth weight, and feeding pattern.

### Association of Maternal Vitamin D Status With Offspring dmft Score and Caries Rate

Maternal 25(OH)D levels (μg/mL) in the third trimester were inversely associated with offspring dmft score (β [SE] = −9.97 [3.97]; *P* = .01), age-standardized dmft score (β [SE] = −16.96 [6.30]; *P* = .01), caries rate (β [SE] = −50.87 [19.78]; *P* = .01), and age-standardized caries rate (β [SE] = −18.50 [6.63]; *P* = .01) after adjustment (eTable 4 in Supplement 1). A significant association was also observed in the second trimester with age-standardized dmft score (β [SE] = −22.69 [8.88]; *P* = .01), while no significant associations were found for the first trimester.

As shown in Table 4, a per-μg/mL increase in maternal 25(OH)D levels was inversely associated with offspring dmft score (second trimester: β [SE] = −2.64 [1.30], *P* = .04; third trimester: β [SE] = −3.95 [1.38], *P* = .004) and caries rate (second trimester: β [SE] = −13.66 [6.55], *P* = .04; third trimester: β [SE] = −20.02 [6.91], *P* = .004). Furthermore, offspring of mothers with VDD in the second trimester had higher dmft scores (β [SE] = 0.08 [0.04]; *P* = .04) and caries rate (β [SE] = 0.41 [0.20]; *P* = .04) compared with those without VDD. Compared with vitamin D sufficiency group (≥30 ng/mL), the vitamin D insufficiency group was associated with higher dmft score (third trimester: β [SE] = 0.09 [0.04]; *P* = .04) and caries rate (third trimester: β [SE] = 0.45 [0.22]; *P* = .04), the VDD group was associated with higher dmft score (first trimester: β [SE] = 0.10 [0.04], *P* = .01; second trimester: β [SE] = 0.09 [0.04], *P* = .04; third trimester: β [SE] = 0.12 [0.05], *P* = .02) and caries rate (first trimester: β [SE] = 0.50 [0.21], *P* = .02; second trimester: β [SE] = 0.45 [0.23], *P* = .045; third trimester: β [SE] = 0.58 [0.26], *P* = .02), and the severe VDD group was associated with higher dmft score (first trimester: β [SE] = 0.10 [0.05]; *P* = .03) and caries rate (first trimester: β [SE] = 0.51 [0.23]; *P* = .03).

**Table 4.** Association of Maternal Vitamin D Status in Different Trimesters With Offspring dmft Score and Caries Rate in Generalized Estimating Equation Models^**a**^

Vitamin D status by trimester^b^	No.	dmft Score		Caries rate	
		β (SE)	*P* value	β (SE)	*P* value
**25(OH)D level, μg/mL**					
First trimester	3279	−2.84 (1.46)	.052	−14.17 (7.57)	.06
Second trimester	2095	−2.64 (1.30)	.04	−13.66 (6.55)	.04
Third trimester	2046	−3.95 (1.38)	.004	−20.02 (6.91)	.004
**2 Categories of 25(OH)D levels**					
First trimester					
Vitamin D sufficiency: ≥20 ng/mL	2521	1 [Reference]	NA	1 [Reference]	NA
VDD: <20 ng/mL	758	0.04 (0.03)	.15	0.21 (0.14)	.14
Second trimester					
Vitamin D sufficiency: ≥20 ng/mL	1702	1 [Reference]	NA	1 [Reference]	NA
VDD: <20 ng/mL	393	0.08 (0.04)	.04	0.41 (0.20)	.04
Third trimester					
Vitamin D sufficiency: ≥20 ng/mL	1543	1 [Reference]	NA	1 [Reference]	NA
VDD: <20 ng/mL	503	0.08 (0.05)	.08	0.39 (0.23)	.09
**4 Categories of 25(OH)D levels**					
First trimester					
Vitamin D sufficiency: ≥30 ng/mL	272	1 [Reference]	NA	1 [Reference]	NA
Vitamin D insufficiency: ≥20 to <30 ng/mL	899	0.08 (0.04)	.06	0.38 (0.21)	.07
VDD: ≥12 to <20 ng/mL	1328	0.10 (0.04)	.01	0.50 (0.21)	.02
Severe VDD: <12 ng/mL	780	0.10 (0.05)	.03	0.51 (0.23)	.03
Second trimester					
Vitamin D sufficiency: ≥30 ng/mL	920	1 [Reference]	NA	1 [Reference]	NA
Vitamin D insufficiency: ≥20 to <30 ng/mL	702	0.02 (0.03)	.40	0.12 (0.15)	.43
VDD: ≥12 to <20 ng/mL	363	0.09 (0.04)	.04	0.45 (0.23)	.045
Severe VDD: <12 ng/mL	110	0.10 (0.08)	.21	0.51 (0.41)	.22
Third trimester					
Vitamin D sufficiency: ≥30 ng/mL	987	1 [Reference]	NA	1 [Reference]	NA
Vitamin D insufficiency: ≥20 to <30 ng/mL	567	0.09 (0.04)	.04	0.45 (0.22)	.04
VDD: ≥12 to <20 ng/mL	379	0.12 (0.05)	.02	0.58 (0.26)	.02
Severe VDD: <12 ng/mL	113	0.12 (0.08)	.13	0.63 (0.41)	.13

Abbreviations: 25(OH)D, 25-hydroxyvitamin D; dmft, decayed, missing, or filled teeth; GEE, generalized estimation equation; NA, not applicable; VDD, vitamin D deficiency.

SI conversion factor: To convert micrograms per milliliter to nanomoles per liter, multiply by 2.496.

^a^ Maternal 25(OH)D levels were analyzed in μg/mL to facilitate the interpretation of regression coefficients.

^b^ Vitamin D status at each trimester was calculated in different models. The GEE models were adjusted for maternal age, smoking status, parity, educational level, body mass index, and gestational age and season of 25(OH)D test as well as offspring gestational age at birth, gender, birth weight, and feeding pattern.

## Discussion

In this prospective cohort study, we observed consistent inverse associations between maternal 25(OH)D levels during pregnancy, particularly in the later trimesters (second and third), and odds of ECCs, dmft scores, and caries rates in offspring. Cox proportional hazards regression models also presented inverse associations for ECC risk throughout all trimesters, with the most robust results during the third trimester. Furthermore, categorical analyses indicated that compared with the vitamin D sufficiency group, the offspring of mothers with lower vitamin D status (vitamin D insufficiency, VDD, or severe VDD) had significantly higher hazards of ECCs, increased dmft scores, and caries rates.

Existing epidemic studies were limited by single-time point 25(OH)D measurements and predominantly European populations.^25,31,32,33,34,35^ The ethnicity-based variations in disparities in vitamin D supplementation effectiveness and metabolism-related genes highlight the need for population-specific investigations.^36,37,38^ Our study filled this gap, as to our knowledge, this is the first large-scale prospective cohort study in China to measure 25(OH)D levels across all trimesters, demonstrating consistent associations between higher maternal vitamin D levels and reduced odds of offspring caries. Moreover, to eliminate the potential association of children’s age with the number and proportion of caries, we conducted a sensitivity analysis that showed these associations were stable after standardizing children’s age at the time of dental examination.

The biological mechanisms underlying the potential protective benefit of maternal vitamin D levels against offspring caries remain unknown. One potential mechanism involves its regulation of calcium and phosphate homeostasis through both genomic and nongenomic pathways, which may provide an optimal ionic environment for enamel and dentin mineralization.^13,14^ Adequate maternal vitamin D levels ensure sufficient mineral supply for fetal tooth development. At the cellular level, ameloblasts and odontoblasts express VDR. Vitamin D directly regulates the expression of key enamel matrix proteins through VDR and promotes the synthesis of calcium-binding proteins essential for crystal formation.^15,16^ Furthermore, vitamin D exhibits immunomodulatory effects, which enhance the production of antimicrobial peptides in oral mucosal tissues and strengthen innate immune defense against cariogenic bacteria to prevent caries.^39,40^ Recent evidence suggests that vitamin D may change the epigenetic regulation of genes involved in tooth development and enhance the barrier function of oral epithelium, providing additional protection against bacterial invasion and acid demineralization.^41^

To date, a series of observational studies on the relationship between maternal vitamin D levels and offspring caries have yielded inconsistent conclusions. Some reports suggested protective associations^19,20^; meanwhile, others reported null findings.^24,42,43^ These discrepancies may be largely attributed to methodological limitations, such as inadequate sample sizes and inclusion of older children in mixed dentition. As ECC is, by definition, a disease of the primary dentition, the presence of permanent teeth in mixed dentition obscures accurate ECC assessment. Furthermore, 2 randomized clinical trials of prenatal vitamin D supplementation have generally reported null findings for offspring dental outcomes^22,23^; these results may reflect suboptimal intervention timing rather than a lack of biological association. Studies demonstrated that 25(OH)D_3_ levels reach a plateau after 3 months of supplementation, indicating that vitamin D supplementation likely needs to be initiated prior to pregnancy to achieve maximum benefits.^44^ Collectively, these results emphasize the importance of initiating vitamin D supplementation early in pregnancy and maintaining adequate status throughout gestation. Specifically, sufficient early-pregnancy vitamin D reserves might prevent mid-pregnancy VDD, while maintaining adequate vitamin D levels in the third trimester might improve maternal-fetal calcium metabolism and reduce neonatal VDD.^27,45^ Therefore, we advocate integrating vitamin D screening and supplementation into routine prenatal care and highlighting the need for personalized strategies to ensure optimal vitamin D levels throughout the critical windows of fetal dental development.

### Limitations

There are study limitations that should be acknowledged. First, several potential confounding factors were lacking, such as offspring vitamin D intake, fluoride toothpaste use, sugar intake, and family caries history, which might influence caries risk. Second, the potential for selection bias exists because of the incomplete measurement of maternal 25(OH)D levels across all 3 trimesters and irregular dental examinations in offspring from age 1 to 6 years. Moreover, progressive loss to follow-up with increasing child age may further introduce selection bias (eTable 1 in Supplement 1). Finally, the sample was drawn from a developed coastal region in China. Future prospective and interventional studies with large samples and measuring maternal vitamin D levels throughout pregnancy are needed to validate the generalizability of our findings.

## Conclusions

In this mother-offspring paired cohort study, higher maternal gestational vitamin D levels, particularly in the mid- to late-trimester, were associated with reduced odds of ECC, lower offspring dmft scores, and caries rate. These findings suggest that vitamin D supplementation during pregnancy and even before conception to maintain sufficient vitamin D status throughout pregnancy may help reduce the risk and severity of childhood dental caries.

## Notes

Supplement 1. **eMethods.** Additional Details **eTable 1.** Follow-Up Status of Offspring by Age Group as of November 1, 2022 **eTable 2.** Caries-Related Characteristics of Offspring at Different Age Assessments **eTable 3.** Comparison of Maternal Vitamin D Status During Pregnancy Between Offspring With and Without Early Childhood Caries (ECCs) **eTable 4.** Association of Maternal 25(OH)D Levels With Offspring dmft and Caries Rate **eFigure 1.** Flowchart Diagram of Inclusion in the Study **eFigure 2.** Restricted Cubic Spline Models of the Association of Maternal 25(OH)D Levels With Offspring dmft and Caries Rate **eFigure 3.** Restricted Cubic Spline Models of the Association Between Maternal 25(OH)D Levels and Offspring ECC

Supplement 2.
Data Sharing Statement
