# Supplementary material for: Vitamin D Levels During Pregnancy and Dental Caries in Offspring
Source: JAMA Netw Open. 2025 Dec 2;8(12):e2546166. doi: 10.1001/jamanetworkopen.2025.46166 (PMC12673410; doi:10.1001/jamanetworkopen.2025.46166)
Supplement: Supplement 1. — eMethods. Additional Details eTable 1. Follow-Up Status of Offspring by Age Group as of November 1, 2022 eTable 2. Caries-Related Characteristics of Offspring at Different Age Assessments eTable 3. Comparison of Maternal Vitamin D Status During Pregnancy Between Offspring With and Without Early Childhood Caries (ECCs) eTable 4. Association of Maternal 25(OH)D Levels With Offspring dmft and Caries Rate eFigure 1. Flowchart Diagram of Inclusion in the Study eFigure 2. Restricted Cubic Spline Models of the Association of Maternal 25(OH)D Levels With Offspring dmft and Caries Rate eFigure 3. Restricted Cubic Spline Models of the Association Between Maternal 25(OH)D Levels and Offspring ECC [file jamanetwopen-e2546166-s001.pdf]

Supplemental Online Content

Xu N, Chen Z, Wang B, et al. Vitamin D levels during pregnancy and dental caries in offspring. *JAMA Netw Open*. 2025;8(12):e2546166. doi: 10.1001/jamanetworkopen.2025.46166

eMethods. Additional Details
eTable 1. Follow-Up Status of Offspring by Age Group as of November 1, 2022
eTable 2. Caries-Related Characteristics of Offspring at Different Age Assessments
eTable 3. Comparison of Maternal Vitamin D Status During Pregnancy Between Offspring With and Without Early Childhood Caries (ECCs)
eTable 4. Association of Maternal 25(OH)D Levels With Offspring dmft and Caries Rate
eFigure 1. Flowchart Diagram of Inclusion in the Study
eFigure 2. Restricted Cubic Spline Models of the Association of Maternal 25(OH)D Levels With Offspring dmft and Caries Rate
eFigure 3. Restricted Cubic Spline Models of the Association Between Maternal 25(OH)D Levels and Offspring ECC

This supplemental material has been provided by the authors to give readers additional information about their work.

© 2025 Xu N et al. *JAMA Network Open*

eMethods. Additional Details

Missing Data Imputation

Missing data were imputed using the 'MissForest' package in R, a non-parametric method based on random forests suitable for mixed data types. The following variables with missing values were included: feeding pattern (0.19%), smoking (1.68%), parity (11.73%), and education (4.26%). All analytic variables were used in the imputation model to preserve multivariate relationships. Single imputation was performed, given the low proportion of missingness.

Generalized Estimating Equations (GEE) Model Specification

In all GEE models, we specified an exchangeable correlation structure to account for within-subject dependence across repeated dental examinations. This structure assumes constant correlation between any two measurements within the same child over time. The models were implemented using the geeglm function from the 'geepack' package in R, with family set to Gaussian for continuous outcomes (dmft and caries rate). The cluster variable (id) was set to subject ID, and the time ordering variable (waves) was specified as child age at examination.

Age standardization of dmft and caries rate

The age-standardization was performed by calculating the mean caries rate for each age group using the entire cohort comprising all individuals with children's oral examination records and complete examination and birth dates (for calculating exact age in months). Subsequently, for the sub-cohort used in the analysis (i.e., the group matched with mothers and meeting the specific inclusion and exclusion criteria), the

© 2025 Xu N et al. *JAMA Network Open*

observed caries rate (caries rate) of each child was divided by the mean caries rate of their corresponding age group derived from the total population. This process generated the standardized caries rate, using the total population's age-specific means as the standard to remove age-related variation from the outcomes of the sub-cohort.

© 2025 Xu N et al. *JAMA Network Open*

eTable 1. Follow-up status of offspring by age group as of November 1, 2022

Age Group (months)	Eligible Participants (n)	Actual Follow-up (n)	Loss to Follow-up (%)
12-23	4109	3949	3.9
24-35	3959	3763	5.0
36-47	3555	3175	10.7
48-59	2811	2058	26.8
60-71	1802	1091	39.5

eTable 2. Caries-related characteristics of offspring at different age assessments

**Variables**	**12-23 month (n = 3949)**	**24-35 month (n = 3763)**	**36-47 month (n = 3175)**	**48-59 month (n = 2058)**	**60-71 month (n = 1091)**
**Age at detal examination, month**	18.0 (18.0-18.0)	30.0 (30.0-31.0)	37.0 (36.0-40.0)	53.0 (51.0-56.0)	65.0 (62.0-69.0)
**dmft among children with ECC**	4.0 (2.0-4.0)	2.0 (2.0-4.0)	2.0 (2.0-4.0)	2.0 (2.0-5.0)	2.0 (2.0-5.0)
**Caries rate among children with ECC, %**	25.0 (15.6-27.1)	11.1 (10.0-20.0)	10.0 (10.0-20.0)	10.5 (10.0-25.0)	10.5 (10.0-25.0)
**ECC**					
no	3939 (99.7)	3596 (95.6)	2713 (85.4)	1353 (65.7)	653 (59.9)
yes	10 (0.3)	167 (4.4)	462 (14.6)	705 (34.3)	438 (40.1)

The table presents cross-sectional summaries at each age point. Data represent repeated longitudinal measurements from the same children across different age groups. The variables are presented as median (IQR) for continuous variables and n (%) for categorical variables. ECC, early childhood caries; dmft, decayed, missing, or filled teeth; caries rate, the proportion of caries in the number of teeth. The dmft and caries rate are reported only for children with ECC, as those without ECC have a dmft of 0 and caries rate of 0%.

© 2025 Xu N et al. *JAMA Network Open*

eTable 3. Comparison of maternal vitamin D status during pregnancy between offspring with and without early childhood caries (ECCs)

Vitamin D Trimester	Group	N	25(OH)D levels (ng/ml)		VDD (%)	
			Median (IQR)	*P*-w	Rate	*P*-chi
first	With ECCs	758	16.8 (12.6-22.5)	.99	64.9	.72
	Without ECCs	2521	17.0 (12.1-23.0)		64.1	
second	With ECCs	393	26.9 (18.8-34.3)	.001	27.5	.01
	Without ECCs	1702	28.7 (21.2-36.1)		21.4	
third	With ECCs	503	26.1 (17.3-34.7)	.001	31.8	.001
	Without ECCs	1543	30.6 (21.4-39.6)		21.5	

P-w: the p-value of the Wilcoxon test; *P*-chi: the p-value of the Chi-square test.

© 2025 Xu N et al. *JAMA Network Open*

eTable 4. Association of maternal 25(OH)D levels with offspring dmft and caries rate[a]

Vitamin D trimester	N	Original outcome[b]		Age-standardized outcome[c]	
		β (SE)	*P*	β (SE)	*P*
**dmft**					
first	3279	-4.06 (4.44)	.36	-3.57 (6.12)	.56
second	2095	-6.12 (3.83)	.11	-22.69 (8.88)	.01
third	2046	-9.97 (3.97)	.01	-16.96 (6.30)	.01
**caries rate**					
first	3279	-20.73 (22.16)	.35	-6.03 (6.09)	.32
second	2095	-31.24 (19.20)	.10	-13.84 (9.01)	.12
third	2046	-50.87 (19.78)	.01	-18.50 (6.63)	.01

[a]Maternal 25(OH)D levels were analyzed in μg/mL to facilitate the interpretation of regression coefficients.

[b]Original dmft and caries rate as the outcome respectively, and adjusted for maternal age, smoking status, parity, education, body mass index, maternal gestational age and season of 25(OH)D test, and offspring gestational age at birth, gender, birth weight, and feeding pattern;

[c]Age-standardized dmft and caries rate as the outcome respectively, adjusted for the same covariates as in the Model above.

© 2025 Xu N et al. *JAMA Network Open*

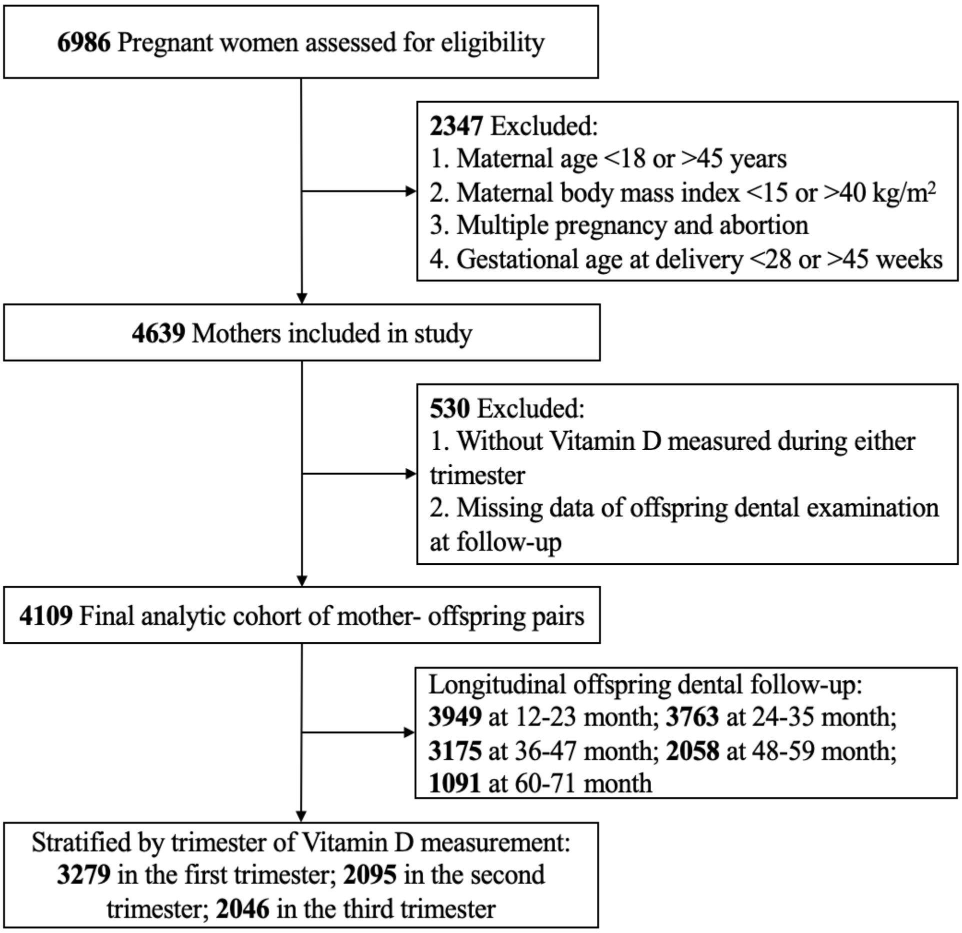

eFigure 1. Flowchart diagram of inclusion in the study

© 2025 Xu N et al. *JAMA Network Open*

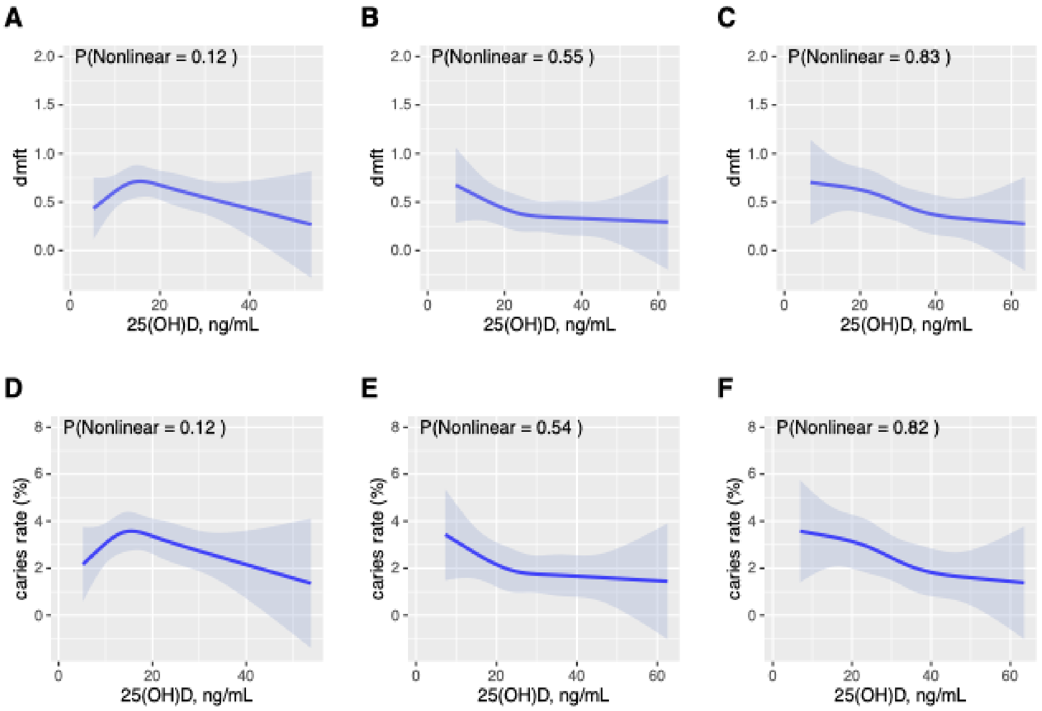

eFigure 2. Restricted cubic spline models of the association of maternal 25(OH)D levels with offspring dmft and caries rate

(A) association between 25(OH)D levels in the first trimester and dmft; (B) association between 25(OH)D levels in the second trimester and dmft; (C) association between 25(OH)D levels in the third trimester and dmft; (D) association between 25(OH)D levels in the first trimester and caries rate; (E) association between 25(OH)D levels in the second trimester and caries rate; (F) association between 25(OH)D levels in the third trimester and caries rate.

adjusted for maternal age, smoking status, parity, education, body mass index, maternal gestational age and season of 25(OH)D test, and offspring gestational age at birth, gender, birth weight, and feeding pattern.

© 2025 Xu N et al. *JAMA Network Open*

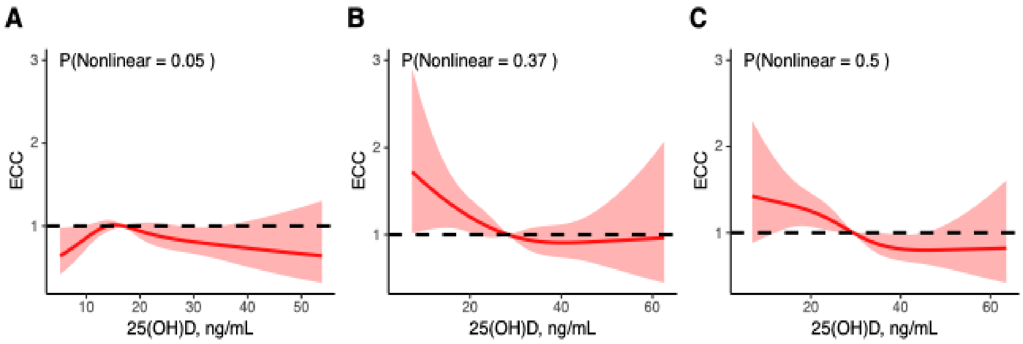

eFigure 3. Restricted cubic spline models of the association between maternal 25(OH)D levels and offspring ECC
(A) association between 25(OH)D levels in the first trimester and ECC; (B) association between 25(OH)D levels in the second trimester and ECC; (C): association between 25(OH)D levels in the third trimester and ECC.
adjusted for maternal age, smoking status, parity, education, body mass index, maternal gestational age and season of 25(OH)D test, and offspring gestational age at birth, gender, birth weight, and feeding pattern.

© 2025 Xu N et al. *JAMA Network Open*
