# Supplementary material for: Vitamin D Levels During Pregnancy and Dental Caries in Offspring
Source: JAMA Netw Open. 2025 Dec 2;8(12):e2546166. doi: 10.1001/jamanetworkopen.2025.46166 (PMC12673410; doi:10.1001/jamanetworkopen.2025.46166)
Supplement: Supplement 2. — Data Sharing Statement [file jamanetwopen-e2546166-s002.pdf]

Data Sharing Statement

Xu. Vitamin D Levels During Pregnancy and Dental Caries in Offspring. *JAMA Netw Open*. Published December 02, 2025. doi:10.1001/jamanetworkopen.2025.46166

Data
Data available: No

Additional Information
Explanation for why data not available: The data presented in this study are available on request from the corresponding author. The data are not publicly available because they contain information that could compromise the privacy of the research participants.
